# Molecular dynamic studies into the comparative optimization of thermo-mechanical characters of nano-composites of Ag and Cu reinforced by Graphene

**DOI:** 10.1371/journal.pone.0269566

**Published:** 2023-02-09

**Authors:** Qaisar Anjam, Nadeem Nasir, Salman Cheema, Zaighum Tanveer, Muhammad Imran, Nasir Amin

**Affiliations:** 1 Department of Physics, Govt. College University Faisalabad (GCUF), Faisalabad, Pakistan; 2 School of Applied Science, National Textile University Faisalabad, Faisalabad, Pakistan; Beijing University of Technology, CHINA

## Abstract

This article fundamentally aims at the comparative study of thermo-mechanical characters of Gr/Ag and Gr/Cu nano-composites. For demonstration purposes, three dimensions that is, (1 0 0), (1 1 0) and (1 1 1), of the metals attached with single layer Graphene sheet are considered. The study is facilitated by the adaptation of the molecular dynamic simulations of the soft LAMMPS to mimic the broad range of experimental environment. The attributes of each structure and their orientations are elaborated over wide range of experimental states, encompassing temperature ranging from 300 K to 1500 K, to assess the melting behavior. The thermal and structural properties are explored by employing mean square displacement (MSD) and radial distribution function (RDF). Furthermore, the mechanical characters are elaborated along both arm-chair and zigzag directions. The findings are supported by producing relevant graphical displays of stress-strain curves and generating extravagant depictions of various dislocations with the application of visual molecular dynamics (VMD) tool. On the basis of intense and careful computational investigations, we witnessed that the Gr/Cu (1 1 1) orientation produced most profound melting characteristics along with distinctive strengthening and fracture mechanism. These outcomes are consistent in comparison of both Gr/Metals layered structures and also with respect to all considered metallic orientations. The findings are discussed thoroughly in a well-structured and synchronized fashion throughout the article.

## 1. Introduction

Since the time of its realization, the utilization of the Graphene (Gr) [1] and its derivatives has bloomed as a focal research area in professional and commercial research circles involved in the field of Gr/Metal nano-composites. The wide appreciation of the novel material is due to the insignias such as, extravagant mechanical properties, exaggerated thermal attributes and substantial optical transmittance [2, 3]. There is no dearth of literature investigating mechanical characteristics of this flat monolayer of carbon atoms bonded with three others, two-dimensional hexagonal honeycomb lattice structure [4, 5]. For example, [6] elaborated the tensile strength of this two-dimensional crystalline structure as a function of Stone-Wales and vacancy defects prevalent in the structure. For more insight, one may consult [7, 8]. Further, [9] studied the buckling strain of the variants of Gr with varying in-plane compressive strains, see also [10]. Moreover, [11, 12] focused on exploring the mechanical characteristics of the material with respect to surface functionalization. They concluded even stronger bond between functionalized Gr and mechanical properties, see also [13–15] for more specifics.

Similarly, the study of thermal characteristics has been topic of numerous investigative efforts. A rich stream of publications envisaged that Gr and its derivatives exhibit excellent thermal features vital for thermal management in electromechanical systems. For example, [16] explored the thermal contraction of Gr in relation with temperature. Furthermore, [17] notified sterling mobility of the two-dimensional material as a natural assistance in conservation and utilization of thermal energy. Moreover, [18, 19] targeted the exploration of thermal conductivity of Gr and Gr coupled materials with respect to variety of parameters. For more elaborative accounts, one may consult [20–22], as well.

Motivated by the inherent characters of Gr, number of researchers have passionately persuaded the inquiry of mechanical and viscoelastic properties of Gr-reinforced composites. For example, improved electric conductivity due to Gr based nano-composite has been topic of [23, 24]. These efforts concluded that the realized composites possess more profound thermal behavior. Furthermore, [25] focused on exploring the mechanical properties of Gr/Metal interface in relation with dislocation gliding. For more interesting details, one may consult [26–28] comprehending the capability of Gr and its derivatives to pin and annihilate dislocations.

These ongoing developments have seen a substantial rise of Gr-based composites usage in wide range of scientific applications such as, preparation of flexible wearable protective gears [29, 30], production of automotive [31, 32], health surveillance [33, 34], intelligent systems development [35, 36] and military applications [37, 38].

**Fig 1:**
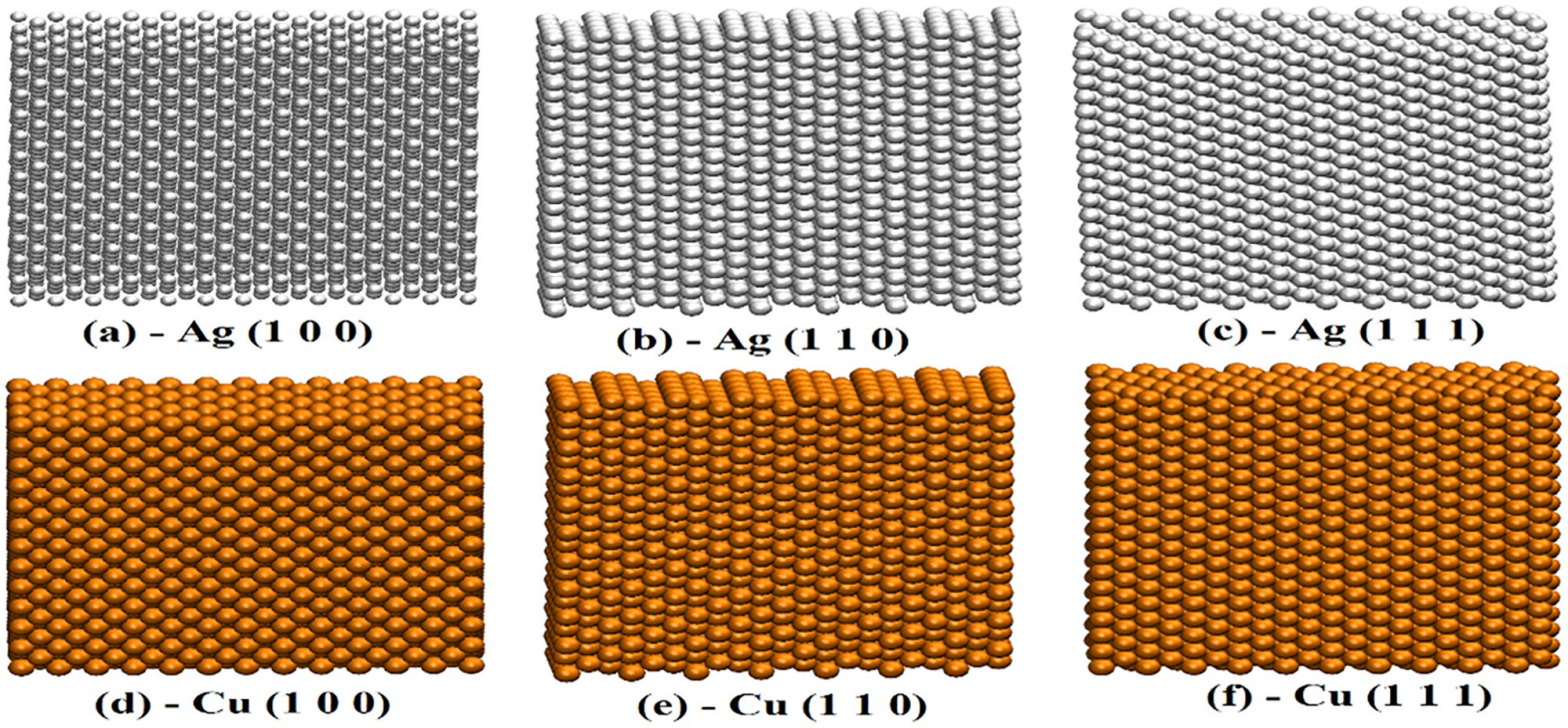
Schematic representation of pure Ag and Cu with respect to different orientations.

Obliging the unique attributes of the Gr, this article primarily focuses on the study of comparative performance of thermo-mechanical properties of Gr/Silver (Gr/Ag) and Gr/ Copper (Gr/Cu) nano-composites. It is to be noted that both contemporaries (Ag and Cu) own interesting characteristics such as notable electric and thermal conductivity, superior mechanical strength, suitable optical reflectivity, flexibility and malleable transition-ability [39–42]. Moreover, simplistic and low-cost fabrication process further advocates the feasibility of these metals in commercial usages. Because of their potentials, one may notice overwhelming utility of Cu [43, 44] and Ag [45, 46] in various forms covering broad range of commercial and professional applications. In this study, single layer Gr stacked over metal substrates (Ag and Cu) is studied. Moreover, the investigation is further enriched by considering three orientations of metal substrates such as, (1 0 0), (1 1 0) and (1 1 1). This comprehensive structure is then exploited for the study of melting behavior using mean square displacement (MSD) and radial distribution function (RDF). Furthermore, dislocations are studied with respect to numbers of carbon atoms in the form of pentagon and heptagon producing glide dislocations. Whereas, octagons are considered to produce shuffle dislocations. On the other hand, the mechanical attributes are explored by drawing stress-strain curves to evaluate hardness, load bearing capacity, ductility and brittleness of the realized materials. Moreover, the aforementioned characters are studied along with arm-chair and zigzag directions. The Fig 1 displays the schematic representation of various pre-considered orientations for pure silver and pure copper.

Although, the experimental procedures remain prime means to enumerate the design outlook of composite materials, however, experimental assessment of multi-dimensional mechanism pose unanticipated challenges [47]. To tackle the hurdle, the usage of computational simulations of composite to mimic the experimental environment have gained well appreciated reputation [48, 49]. The study adopts the molecular dynamic (MD) simulations soft of LAMMPS to investigate the interfaces of Gr with Ag and Cu while considering wide range of parametric setting. To delineate the popularity of the MD simulation methods, Table 1 below presents some of the ongoing research efforts using MD simulation to explore the characters of Gr-oriented substances.

**Table 1:** Ongoing modeling efforts with respect to Graphene-oriented substrates.

Graphene Type	Graphene Layer	Modeling Technique
Unmodified, vacancy defective	Single	MD [50]
Na functionalized	Single	MD/ab initio [51]
Unmodified/Armchair, Zigzag & vdW	Single	MD [52]
Amine/carboxyl/hydroxyl and methyl functionalised	Single	MD/LAMMPS [53]
Unmodified/nano-ribbon (GNR)	Single	MD [54]
Graphitic Carbon Nitride/Armchair, zigzag	Single	MD/LAMMPS [55]
Hybrid graphene/TiO2	Single	MD [56]
Unmodified/ Silicon functionalised/ Armchair, zigzag	Single	MD/LAMMPS [57]
Unmodified/Carboxylic modified	Single	MD/LAMMPS [58]
Hydroxyl, fluorine, Amine/TETA functionalised	Single	MD/LAMMPS [59]
Non-covalent functionalisation Pyrenebutyl, Pyrenebutyric acid, 1-pyrenebutylamine	Single	MD/LAMMPS [60]
Hydrogen, Hydroxyl, Amine and Methyl functionalized	Single	MD [61]
Graphene (Fullerene)	Single	MD [62]
Unmodified (different chiralities)	Single	MSM and MD/LAMMPS [63]
Unmodified (S-W defective)	Single	MD/LAMMPS [64]
Unmodified	Single	MD/DL-Poly [65]
Unmodified	Double	MD [66]
Hydrogen functionalized	Double	MD/QM [67]
Unmodified	Double	MD/LAMMPS [55]
Amine functionalized	Three	MD [68]
Oxide functionalized	Multiple	MD [69]
Unmodified/Nano-ribbon	Multiple	MD/Non-local Timoshenko Model [70]
Rippled Graphene/ Armchair, zigzag	Multiple	MD [71]
Unmodified	Multiple	MD/Numerical Method [72]
Unmodified/Iron oxide	Multiple	MD /FEM [73]
Graphene (nano-ribbons)	Multiple	MD/LAMMPS [74]
Disk shape graphite nano-platelet	Multiple	MD [75]
Unmodified	Single/Four	MD/micromechanics [76]
Unmodified (vacancy defected)	Single, Multiple	FEM (ANSYS) /MD [77]

This article has been divided into four prime partitions. The next section (Section 2) documents the modelling strategy by introducing various parametric settings launched to execute the simulation operations. In section (3), documents the major outcomes along with satisfying discussions of the results. Lastly, section (4) summarizes the conducted research efforts along with few future research prospects.

## 2. Methods and materials

### 2.1 Preliminaries

This research fundamentally aims at the study of thermal characters along with mechanical attributes of the Gr/Metals nano-composites. The objectives are attained by the launch of MD simulations soft of LAMMPS using appropriate and well cherished potential functions including, Embedded Atom Method, Reactive Empirical Bond Order and Lennard-Jones. The melting behaviors as a function of temperature and pressure of the prescribed nano-composites are explored with the applications of MSD. Which is further substantiated by employing RDF highlighting the structure of amorphous alloy. Furthermore, mechanical characters are explored by proceeding the dislocations with respect to numbers of carbon atoms in the form of pentagon, heptagon and octagons. The findings are further elaborated by producing stress-strain curves encapsulating various attributes such as, hardness, load bearing capacity, ductility and brittleness of the realized materials.

### 2.2 Model

**Fig 2:**
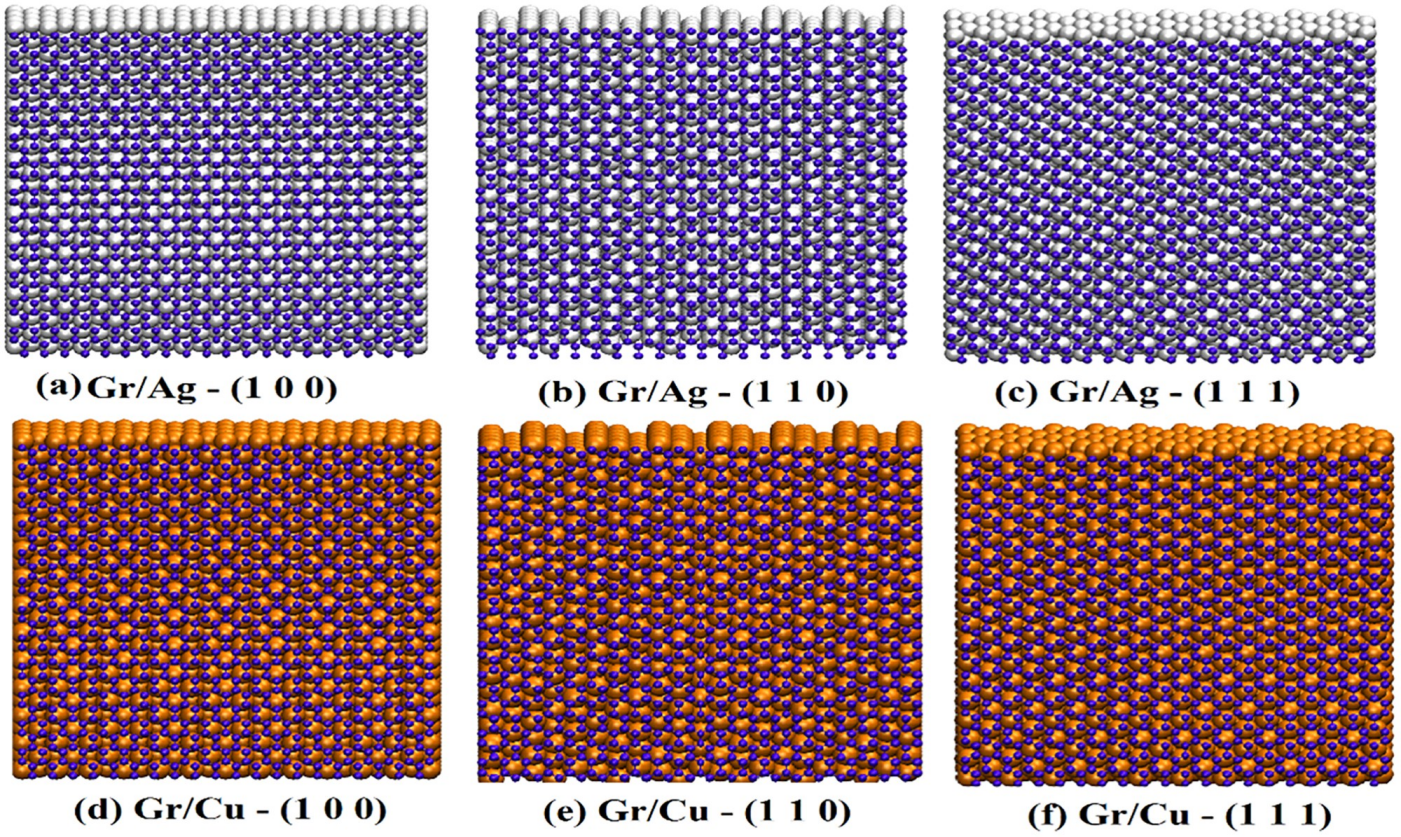
Schematic representation of the Gr/Metal substrate with respect to all orientations.

The MD simulation of the soft LAMMPS is adopted to explore the embedded behavior of Gr/Ag and Gr/Cu. Firstly, a stable configuration of two-dimensional monolayer Gr structure is stacked over Ag and Cu substrates. For the purpose of comparative investigation of thermo-mechanical properties of Gr/Ag and Gr/Cu, size of cell was considered as (40Å, 40Å, 18.3Å) with 1264 atoms loading for both metals. Whereas, size of Gr sheet was set at (40Å, 40Å, 3.4Å) containing 627 atoms. Moreover, the lattice constants for Ag and Cu were taken as 4.09Å and 3.615Å, respectively, whereas Gr lattice constant was set to be 2.46Å. To maintain the generality of the research, considered three orientations of the metals such as (1 0 0), (1 1 0) and (1 1 1). The schematic representation of the embedded structures with respect to both metals are given in Fig 2, where upper panel provides the display of Gr/Ag realizations and lower panel represents Gr/Cu stacks.

### 2.3 Computational details

The equilibrium state at the time step of 0.001ps was achieved by heating both the metals over the range of 10K to 300K. The melting dynamics of the designed samples were explored by varying the temperature over the range of 300K to 1500K with a jump of 100K, whereas, the simulation steps were restricted as 600,000. The simulation employs periodic boundary conditions (P, P, P) in all directions to overcome the fixed wall effect throughout the performance [78]. The mechanical settings were imposed by employing uniaxial tension loading [79], where, three pre-described models for each metal with varying orientations are constructed. Furthermore, all the samples remained subject to tension loading along with arm-chair and zigzag directions. The uniaxial tension loading under a constant strain rate of 5×10^9^ along the arm-chair direction and zigzag direction are applied.

### 2.4 Potential functions

In this study, well acknowledged potentials are employed to proceed the inquiry. This study used Embedded Atom Method (EAM) potential [78, 79] to describe interatomic interactions of Ag-Ag and Cu-Cu. Further, second generation Reactive Empirical Bond Order (REBO) potential [67] for elaborating the Gr-Gr interactions is involved. Lastly, Lennard-Jones (LJ) potential [73] was launched to evaluate the force between Gr/Ag and Gr/Cu. The total energy of the system is now defined as;

$${\text{E}}_{\text{Total}}={\text{E}}_{\text{Ag},\text{Cu}}{}^{\text{EAM}}+{\text{E}}_{\text{Gr}}{}^{\text{REBO}}+{\text{E}}_{\text{Gr}/\text{Ag},\text{Gr}/\text{Cu}}{}^{\text{LJ}},$$ (1)

where, EAM is written as a function of pair-wise interatomic interactions and embedding energy, such as;

$${\text{E}}^{\text{EAM}}=\underset{\text{i}}{\sum }\left[\frac{1}{2}\sum_{\text{j}\neq \text{i}}\Phi \left({\text{r}}_{\text{ij}}\right)+\text{F}\left({\bar{\rho }}_{\text{i}}\right)\right].$$ (2)

Here, ${\bar{\rho }}_{i}=\sum_{j\neq i}\rho \left(r_{ij}\right)$ and Φ denotes pair potential related to the atom separation, *r*_*ij*_. Also, *F* highlights the embedding energy associated with density ${\bar{\rho }}_{i}$, where *ρ* is atomic density function. The improved and more accurate second generation REBO potential is given as;

$${\text{E}}^{\text{REBO}}=\sum_{\text{i}}\sum_{\text{j}>\text{i}}\left[{\text{V}}_{\text{R}}\left({\text{r}}_{\text{ij}}\right)-{\text{B}}_{\text{ij}}{}^{*}{\text{V}}_{\text{A}}\left({\text{r}}_{\text{ij}}\right)\right],$$ (3)

where, *B*_ij_* indicates bond-order parameter. Also, *V*_*R*_(*r*_*ij*_) is interatomic repulsion parameter with *V*_*A*_(*r*_*ij*_) remains the interatomic attraction parameter [79]. Further, the LJ potential is exercised to model the interactions of Gr/Ag and Gr/Cu such as;

$${\text{E}}^{\text{LJ}}=4\varepsilon \left[{\left(\frac{{\text{r}}_{\text{ij}}}{\sigma }\right)}^{-12}-{\left(\frac{{\text{r}}_{\text{ij}}}{\sigma }\right)}^{-6}\right],$$ (4)

where, the parameters *ε* and *σ* describe the energy scale and collision diameter, respectively [76]. The executing setting of these parameters, including potential depth, equilibrium separation and the cutoff for both composites Gr/Ag and Gr/Cu is given in Table 2 below.

**Table 2:** The pair potential parameters between Gr and metal substrates.

*Gr*/*Ag*			*Gr*/*Cu*		
*ε*_*Gr*/*Ag*_	*σ*_*Gr*/*Ag*_	*r_c_*	*ε*_*Gr*/*Cg*_	*σ*_*Gr*/*Ag*_	*r_c_*
0.0301 *ev*	3.006Å	2.5*σ*	0.02578 *ev*	3.0825Å	2.5*σ*

The conjugate gradient algorithm was employed in order to optimize the models for the attainment of minimized energy system. To further relax the samples, isothermal-isobaric (NPT) ensemble was used by considering a Nose-Hoover thermostat [80]. The visualization investigation is carried out by using visual molecular dynamics (VMD) tool [25].

## 3. Results and discussion

### 3.1 Comparative analysis of melting behavior

This section delineates the melting behavior of Gr/Metal nano-composites. The thermal characters of the materials are studies across all three pre-defined orientations. Moreover, the melting attitude is explored by using MSD and RDF methods. The applicability of MSD in understanding the interatomic interactions in relation to the distance is well documented. Similarly, the utility of RDF in the study of structural stability of substances is well appreciated.

Table 3 below compiles the values of melting temperatures for Gr/Metal nano-composites along with their orientations. The relative percentage changes in melting temperatures in pair-wise comparison manner of the realized materials are provided. The diagonal values of percentage changes are calculated such as, %*change* = ((*Column values*/*Row values*)) × 100, whereas off-diagonals are the inverse of the above given expression. One may immediately notice that the melting behavior of the materials is orientation sensitive. The highest value of temperature for (1 1 1) dimension (1250K) when studying the Gr/Ag is observed. This value shows 5% increase in melting temperature with respect to (1 0 0) and 8% increase while comparing with (1 1 0) orientation. Similarly, in the case of Gr/Cu, the (1 1 1) orientation again noticeably distinct itself from other composites (1332K). The melting temperature of (1 1 1) formation shows 11% increase with respect to (1 0 0) orientation, whereas increment is estimated as 26% while comparing with (1 1 0). In general, one may conclude that Gr/Cu (1 1 1) projects more profound thermal characters.

**Table 3:** Melting temperatures and relative percentage changes in melting behavior with respect to pre-considered orientations.

	*Gr/Ag*			*Gr/Cu*		
	(1 0 0)	(1 1 0)	(1 1 1)	(1 0 0)	(1 1 0)	(1 1 1)
*Melting Temp*.	1190K	1150K	1250K	1191K	1053K	1332K
(1 0 0)	----	4%↓	5%↑	----	12%↓	11%↑
(1 1 0)	4%↑	----	8%↑	12%↑	----	26%↑
(1 1 1)	5%↓	8%↓	----	11%↓	26%↓	----

**Fig 3:**
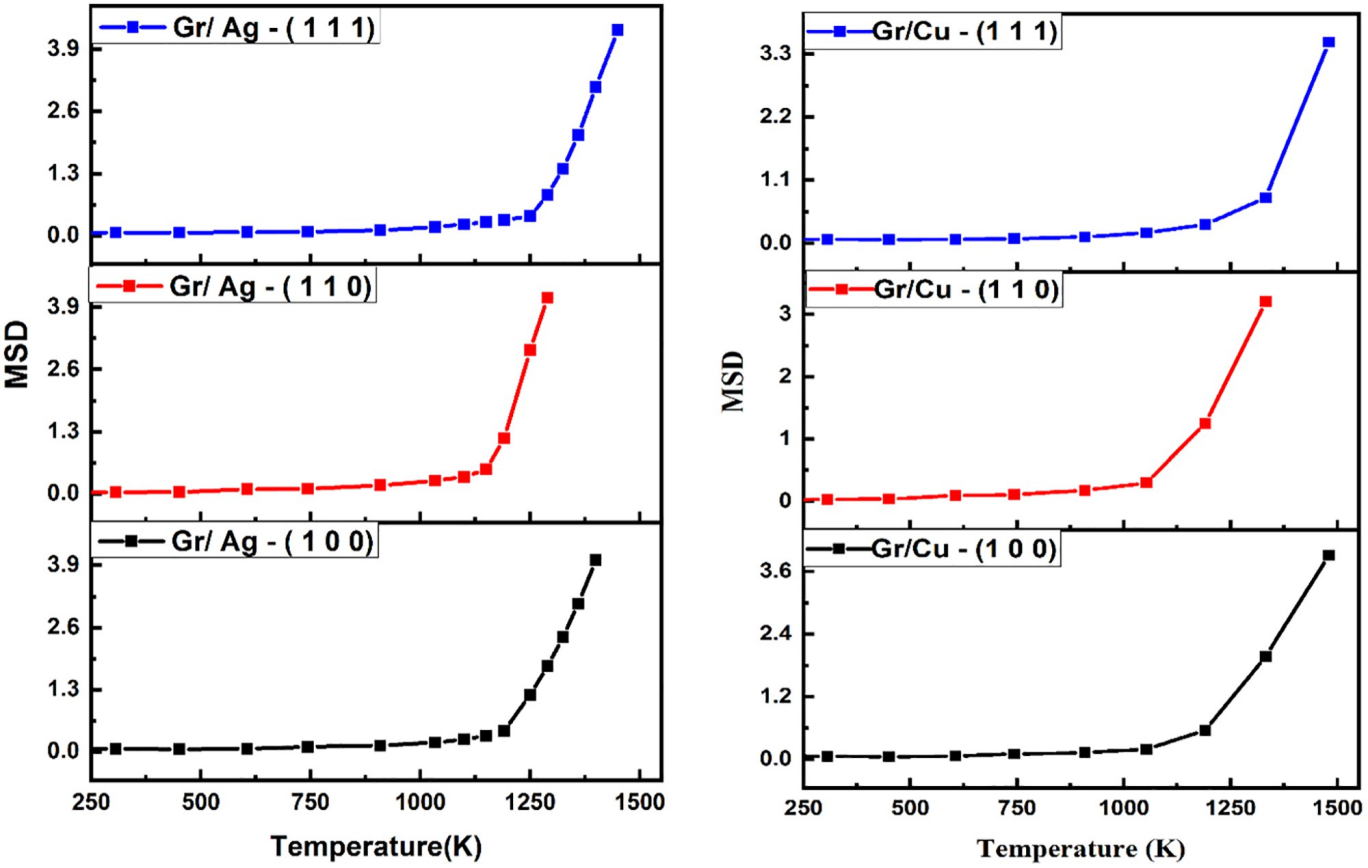
Mean square displacement *vs* varying levels of temperature of Gr/Metals layered structures with respect to all considered orientations.

**Fig 4:**
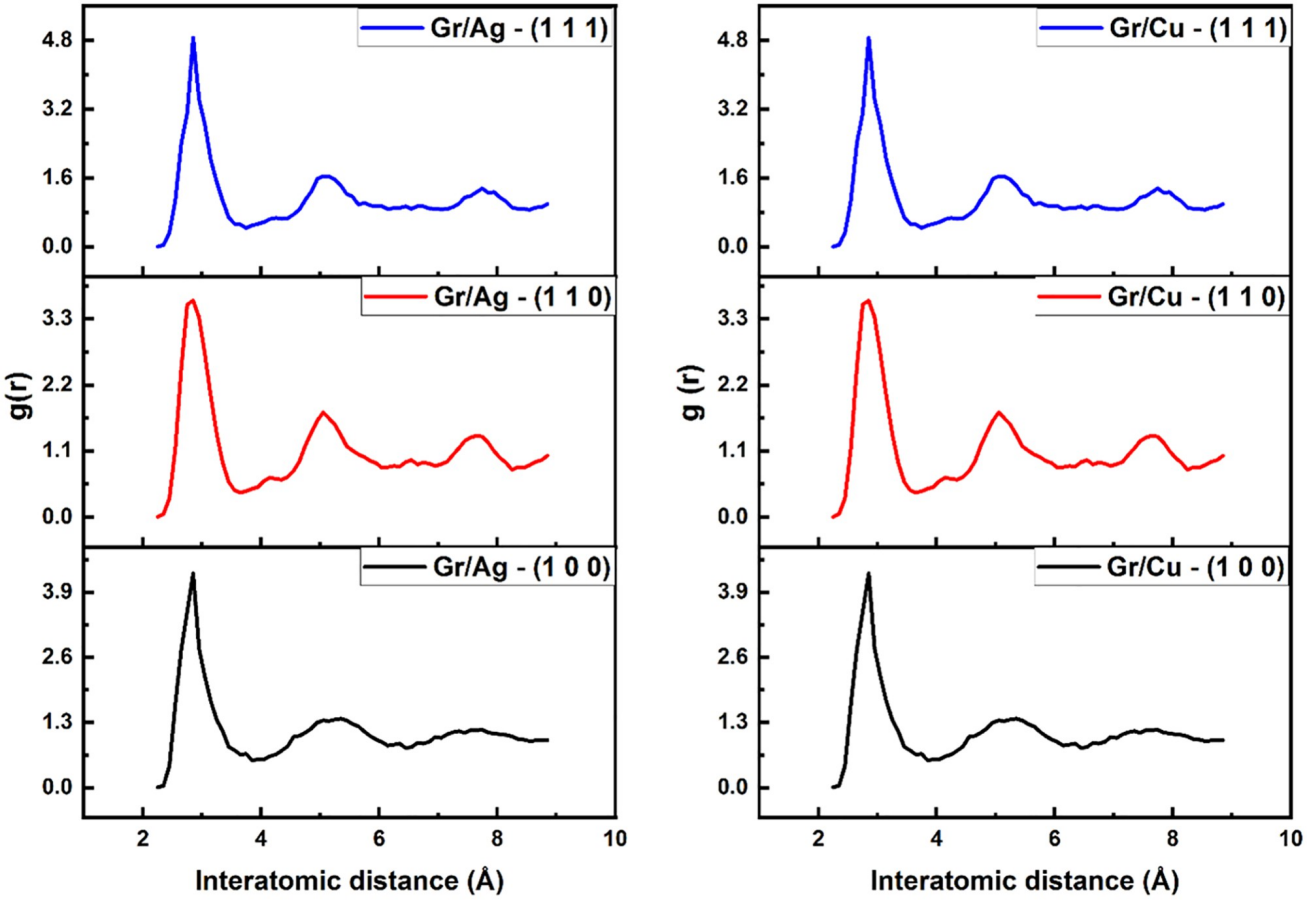
Radial distribution function relating interatomic distance of Gr/Metals layered structures with respect to all considered orientations.

**Fig 5:**
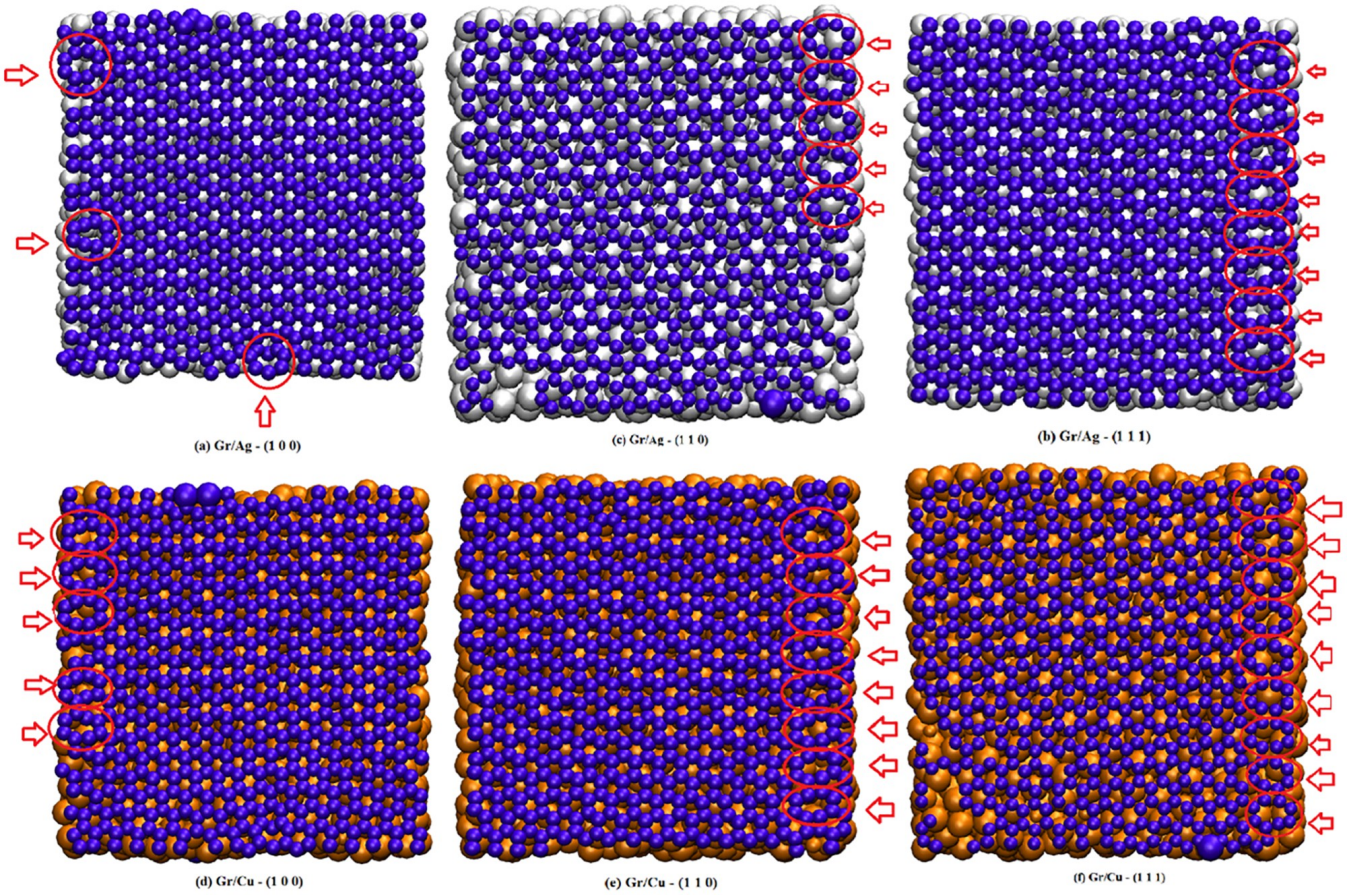
Melting behavior of Gr/Metals layered structures with respect to all considered orientations over the range of 300K to 1500K. The arrowed points indicate the existence of structures specifying the dislocations.

Various thermal aspects of the Gr/Metal nano-composites are displayed in below figures. The Fig 3 quantifies the atomic mobility of contemporary layered structures during equilibration (10K) and heating (300K – 1500K). It remains noticeable that the dimension (1 1 1) projects more profound melting behavior as compared to others dimensions, regardless of the Gr-metallic composition. Moreover, while comparing (1 1 1) orientation across Gr/Ag and Gr/Cu, the copper-oriented material outperforms the silver. The investigation of thermal characters is further elaborated through radial distribution function presenting the structural stability of the Gr-based structures with respect to interatomic distance in Fig 4. The stability of layered structure with (1 1 1) orientation is evident from the distinctive peaks of the plotted functions revealing stronger interfacial interactions. The diminishing peaks indicate the inverse relationship existent between temperature and radial distribution function. Lastly, Fig 5 provides the propensity of dislocations observant in the Gr layers as a result of unsatisfied and dangling bonds prevalent in Gr layer with the increase in temperature. The emergence of dangling bond facilitates the occurrence cross linkage to broken hexagonal ring of Gr which then increases with increase in temperature leads causing higher degree of hardness [81]. The shuffled dislocations are indicated by highlighting the octagon formation observable in the layered structures. The structural stability of the (1 1 1) dimension is sealed by revealing highest number of dislocations associated with both Gr/Metal layered structures. Further, Gr/Cu (1 1 1) shows more extravagant dislocations when compared with the Gr/Ag (1 1 1). The findings of these synchronized investigations, unanimously point towards the superiority of Gr/Cu (1 1 1) composite when thermal characteristics are under study.

### 3.2 Comparative analysis of mechanical behavior

This section is dedicated to the study of mechanical strength of the Gr/Metals layered structures. Further, the mechanical behavior of both materials is enumerated along with arm-chair direction and zigzag direction. The study produces stress-strain curves of the Gr/Ag and Gr/Cu nano-composites reinforced by Gr edges and interface during deformation which then propagate the dislocations.

#### Deformation along arm-chair direction

Table 4 comprehends the numerical values of stress, strain and their percentage changes with respect to all orientations. The maximum value of stress is witnessed as 22.4 G Pa, while exploring the Gr/Ag nano-composite with (1 1 1) orientation. This value shows 13.1% increase in stress bearing as compared to (1 0 0) orientation. Whereas, the 2.2% increase is witnessed when comparing with (1 1 0) orientation. Similarly, in the case of strain evaluation, (1 1 1) orientation outperforms the other contemporaries with value of 0.36. This is 33.3% incremented value of strain in comparison to the (1 0 0) and 20% in case of (1 1 0) orientation.

**Table 4:** Stress and strain summaries for Gr/Metals nano-composites in arm-chair direction.

	*Gr/Ag*			*Gr/Cu*		
	(1 0 0)	(1 1 0)	(1 1 1)	(1 0 0)	(1 1 0)	(1 1 1)
*Stress (G Pa)*	19.8	21.9	22.4	21.9	22.5	26.8
*%age change*	----	10.6%↑	13.1%↑	----	2.6%↓	19.1%↑
	10.6%↓	----	2.2%↑	2.6%↑	----	22.4%↑
	13.1%↓	2.2%↓	----	19.1%↓	22.4%↓	----
*Strain*	0.27	0.30	0.36	0.27	0.30	0.37
*%age change*	----	11.1%↑	33.3%↑	----	11.1%↑	37%↑
	11.1%↓	----	20%↓	11.1%↓	----	23.3%↑
	33.3%↓	20%↓	----	37%↓	23.3%↓	----

Next comprehend are the stress-strain behavior of the Gr/Cu nano-composites. The orientation prepared as (1 1 1) consistently outperforms the rival composites. In case of stress, maximal value of 26.8 G Pa related with the (1 1 1) orientation is estimated. This value highlights 19.1% increase as compared to (1 0 0), whereas it remains 22.4% higher in comparison of (1 1 0) orientation. The study of strain again advocates the use of (1 1 1) orientation with maximum value of 0.37. This value is distinct in profoundness to the extent of 37% increase as compared to (1 0 0) and 23.3% in comparison of (1 1 0) orientation.

While comparing the overall behavior of Gr/Ag and Gr/Cu materials, one may notice that the Gr/Cu emerges as the more promising material than Gr/Ag. The highest value of stress bearing (26.8 G Pa) and maximum of the strain values (0.37) are associated with Gr/Cu (1 1 1) orientation in comparison to the all-nominated samples.

**Fig 6:**
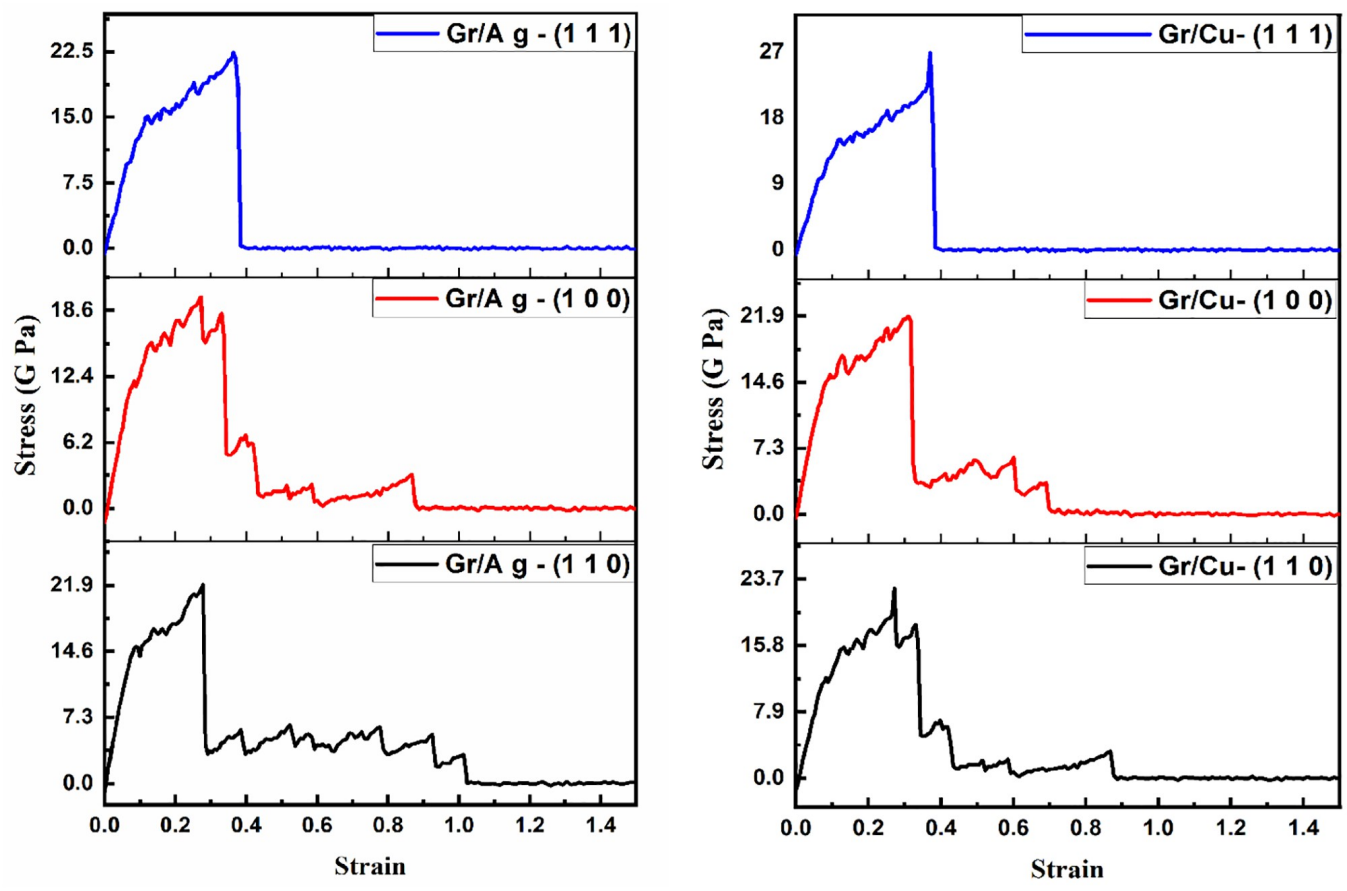
Stress-strain display of Gr/Metals layered structure employing uniaxial tensile loadings along arm-chair direction at varying strain rates.

The above documented numerical findings are further supported by providing stress-strain curves in the Fig 6 with respect to uniaxial tensile loadings along arm-chair direction. The left panel of the figure envisaged the Gr/Ag layered outlook, whereas right panel projects Gr/Cu material, encompassing all the considered domains. It is to noted that, with in each layered structure, considered orientations of metals project distinctive display of the stress-strain curves. For example, the deformities in (1 0 0) are more noticeable and therefore stays least reliable structure as compared to the remaining two dimensions in both Gr/Metals nano-composites that is Gr/Ag and Gr/Cu. Moreover, the (1 1 1) orientation notifies the most convincing mechanical behavior across both Gr-based layered structures. The comparative analysis of both Gr/Ag (1 1 1) and Gr/Cu (1 1 1) reveals that the Gr/Cu (1 1 1) outperforms the contemporary candidate. These findings are consistent with the numerical results comprehended in the Table 4.

**Fig 7:**
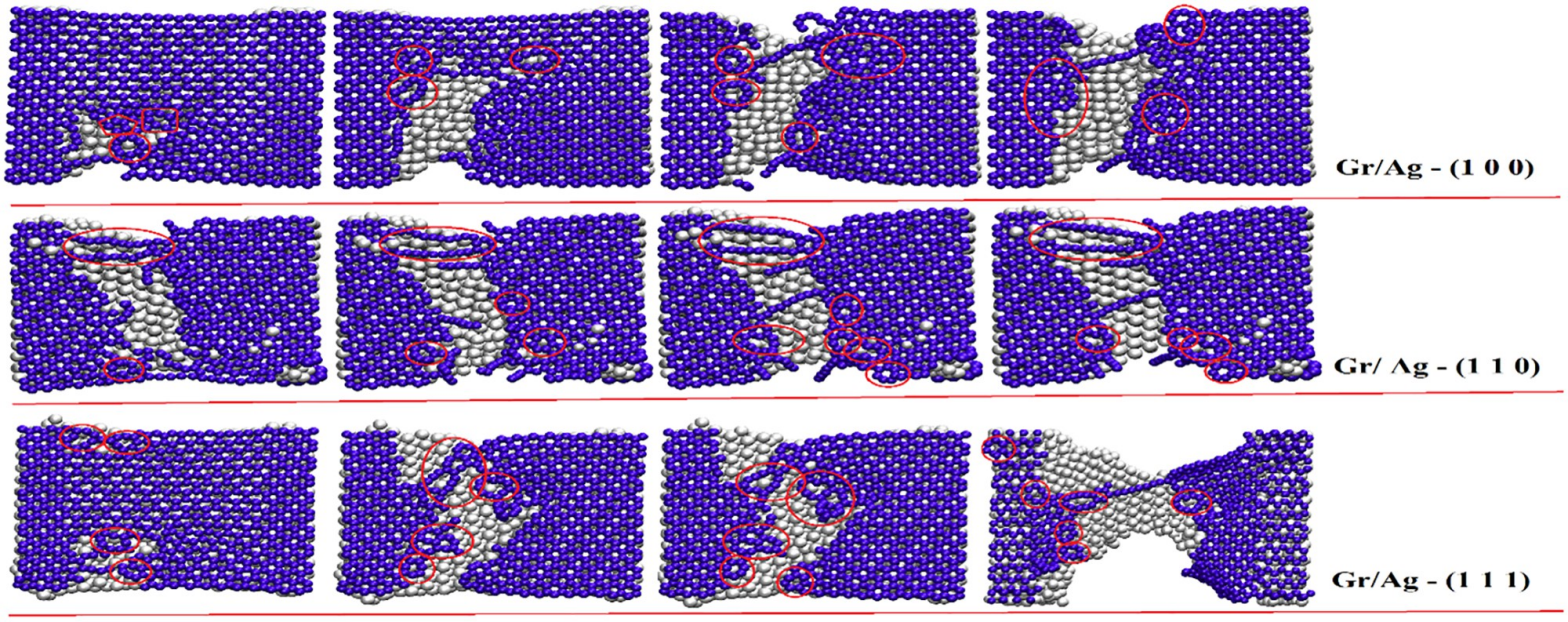
The mechanical characters of the Gr/Ag nano-composites along arm-chair directions with respect to all three orientations. The circles highlighted various types of dislocations.

**Fig 8:**
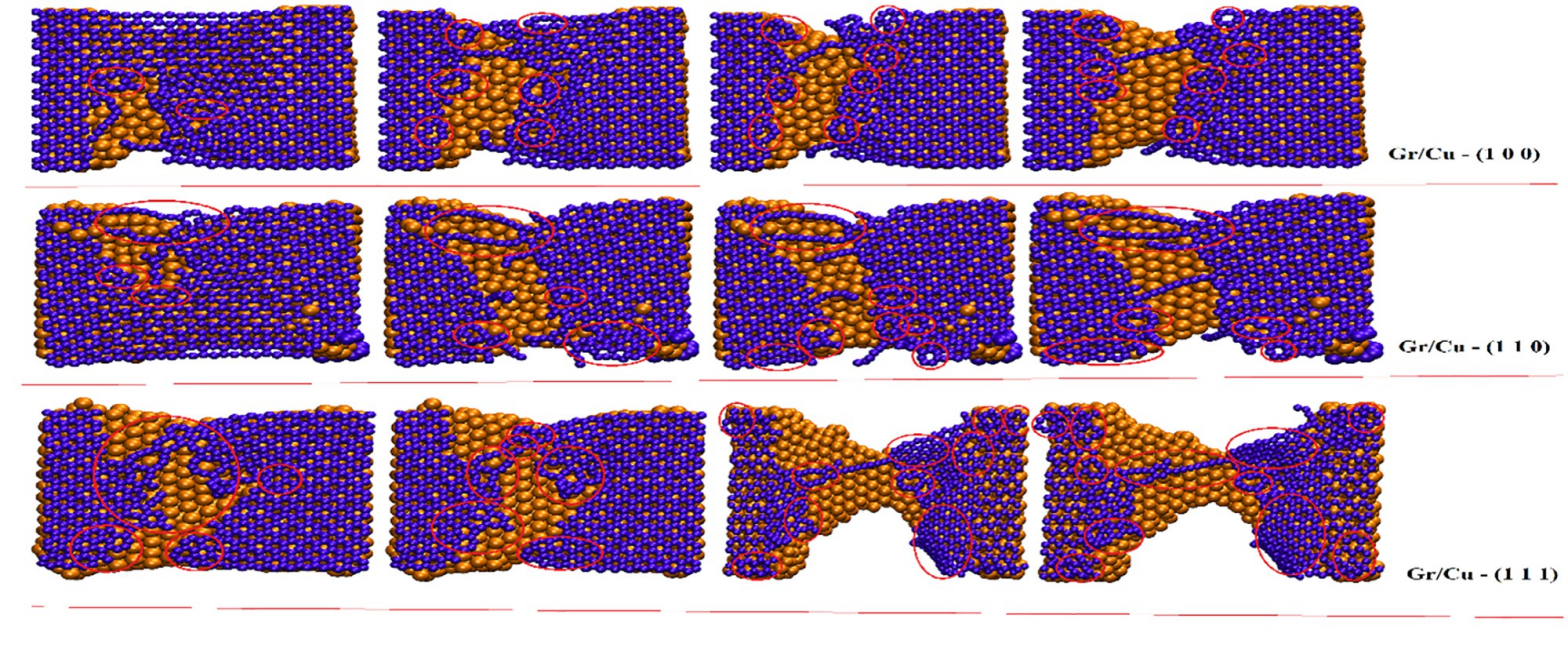
The mechanical characters of the Gr/Cu nano-composites along arm-chair directions with respect to all three orientations. The circles highlighted various types of dislocations.

The investigation into the deformation and strengthening mechanism in both Gr/Ag and Gr/Cu nano-composites reinforcement along arm-chair direction is further summarized in Figs 7 and 8. The objectives are facilitated by employing visual molecular dynamics (VMD) tool. The Fig 7 depicts the fracture process of Gr/Ag nano-composites with respect to all considered orientations of metal nano-structures. The produced snapshots are fetched at different time steps at constant strain rate of 5 × 10^9^, where stress is considered as a function of strain. It is noteworthy that the production of glide dislocations as a result of heptagon and pentagon structures as well as the shuffled dislocations noticeable due to the octagon formations. Also, pinned dislocations remain observable where more numbers of carbon atoms of Graphene break and new bonds emerge randomly. It is to be noted that the breakage of Ag atoms may considered as the outcome of brittle behavior. Various types of dislocations are highlighted by circling them in the relevant snapshots. The Fig 7 clearly indicates the superior mechanical characters Gr/Ag (1 1 1) dimension in comparison with the remaining rival orientations.

Next, Fig 8 comprehends the mechanical attributes of the Gr/Cu nano-composites along arm-chair direction and by taking all afore-documented orientations into consideration. The strengthening attitude remains comprehendible along similar lines as those of Gr/Ag material. Again, while comparing with other contemporary dimensions Gr/Cu (1 1 1) outperforms the competitors.

Lastly, the intra- Gr/Metals comparison highlight the superiority of the mechanical behaviors of the Gr/Cu (1 1 1) nano-composites among all competitors along arm-chair directions. The vividly more numbers of produced dislocations observable in the snapshots verify the advocated characteristic. These findings are profound match with the numerical findings subsequently reported in above sections.

#### Deformation along zigzag direction

Table 5 comprehends the numerical values of stress, strain and their percentage changes with respect to all orientations in zigzag direction. While exploring the Gr/Ag nano-composite, the maximum value of stress is estimated to be 24.5 G Pa, along with (1 1 1) orientation. This value shows 4.8% increase in stress bearing as compared to (1 0 0) orientation. Whereas, the 28.9% increase is witnessed when comparing with (1 1 0) orientation. Next, in the evaluation of strain, again (1 1 1) orientation standout as compared to the other counterparts with a value of 0.42. This value is at 55.5% incremental points of strain in comparison to the (1 0 0) and (1 1 0) orientation.

**Table 5:** Stress and strain summaries for Gr/Metals nano-composites in zigzag direction.

	*Gr/Ag*			*Gr/Cu*		
	(1 0 0)	(1 1 0)	(1 1 1)	(1 0 0)	(1 1 0)	(1 1 1)
*Stress (GPA)*	23.4	19.0	24.5	25.4	21	31
*%age change*	----	18.8%↓	4.7%↑	----	17.3%↓	22.1%↑
	18.8%↑	----	28.9%↑	17.3%↑	----	47.6%↑
	4.7%↓	28.9%↓	----	22.1%↓	47.6%↓	----
*Strain (Unit)*	0.27	0.27	0.42	0.27	0.27	0.39
*%age change*	----	----	55.5%↑	----	----	44.4%↑
	----	----	55.5%↑	----	----	44.4%↑
	55.5%↓	55.5%↓	----	44.4%↓	44.4%↓	----

Next presented are the stress-strain behavior of the Gr/Cu nano-composites. The sample constructed under (1 1 1) orientation again shows superlative characters as compared to other orientations. In case of stress, maximal value of 31 G Pa associated with the (1 1 1) orientation is observed. This value highlights 22.1% increase as compared to (1 0 0), whereas it remains 47.6% higher while comparing with (1 1 0) orientation. The study of strain again advocates the use of (1 1 1) orientation with maximum value of 0.39. This value is distinctive in profoundness to the extent of 44.4% increase as compared to (1 0 0) and (1 1 0) orientation. The overall comparison of the contending materials considering all samples of both Gr/Metals nano-composites, one can vividly notice that the Gr/Cu emerges as the more promising material than Gr/Ag.

**Fig 9:**
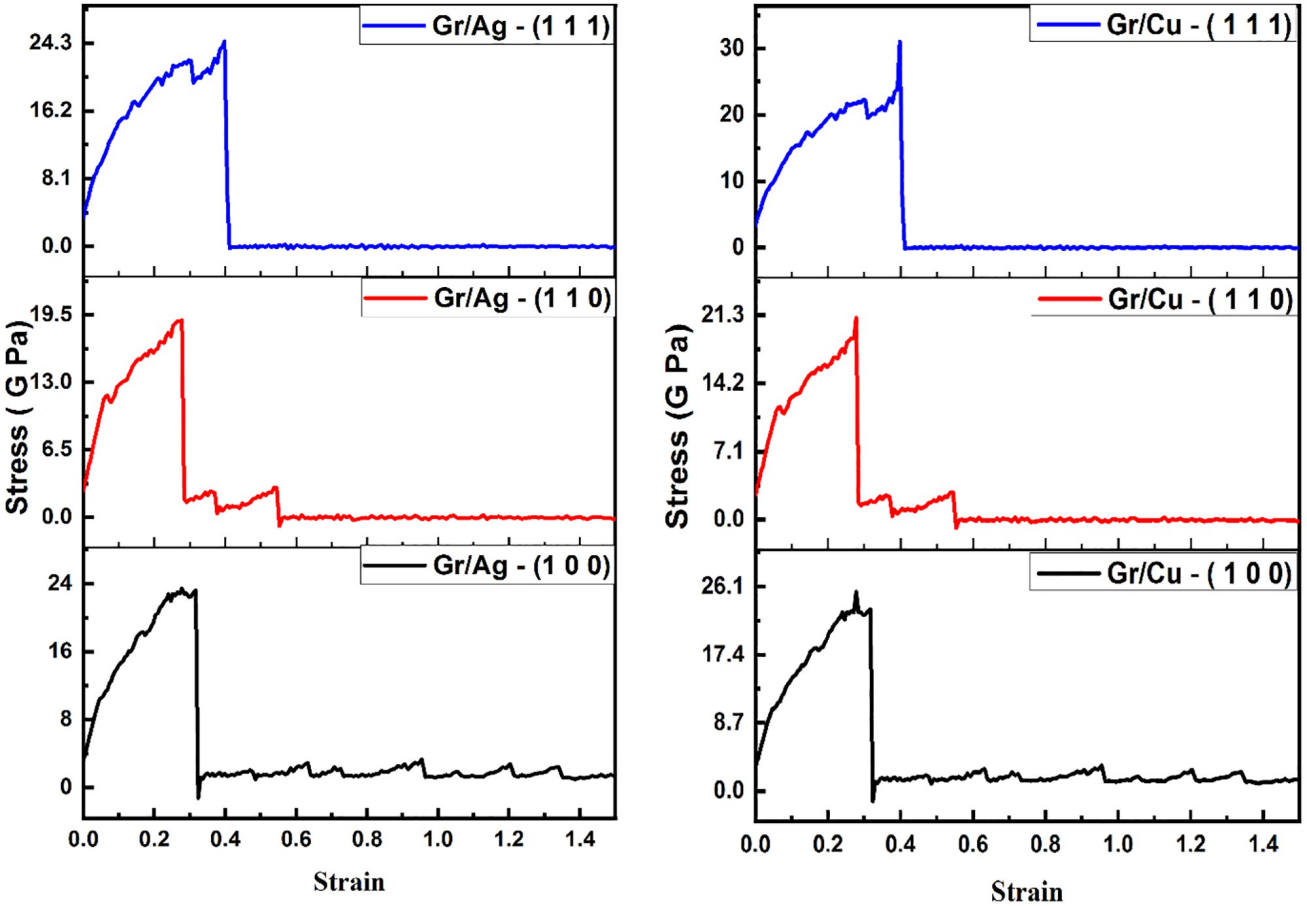
Stress-strain display of Gr/Metals layered structure employing uniaxial tensile loadings along zigzag direction at varying strain rates.

The tabulated outcomes are further elaborated through stress-strain curves projecting relative mechanical attitude with the application of uniaxial tensile loadings along zigzag direction in Fig 9. The superiority of (1 1 1) dimension across both Gr/Metals layered structures is noticeable. Furthermore, Gr/Cu nano-composites (1 1 1) orientation stands itself at the top in the performance hierarchy. The reliability of the Gr/Cu (1 1 1) dimensional material when uniaxial tensile loading is employed is vivid as compared to its close competitor that is Gr/Ag (1 1 1).

**Fig 10:**
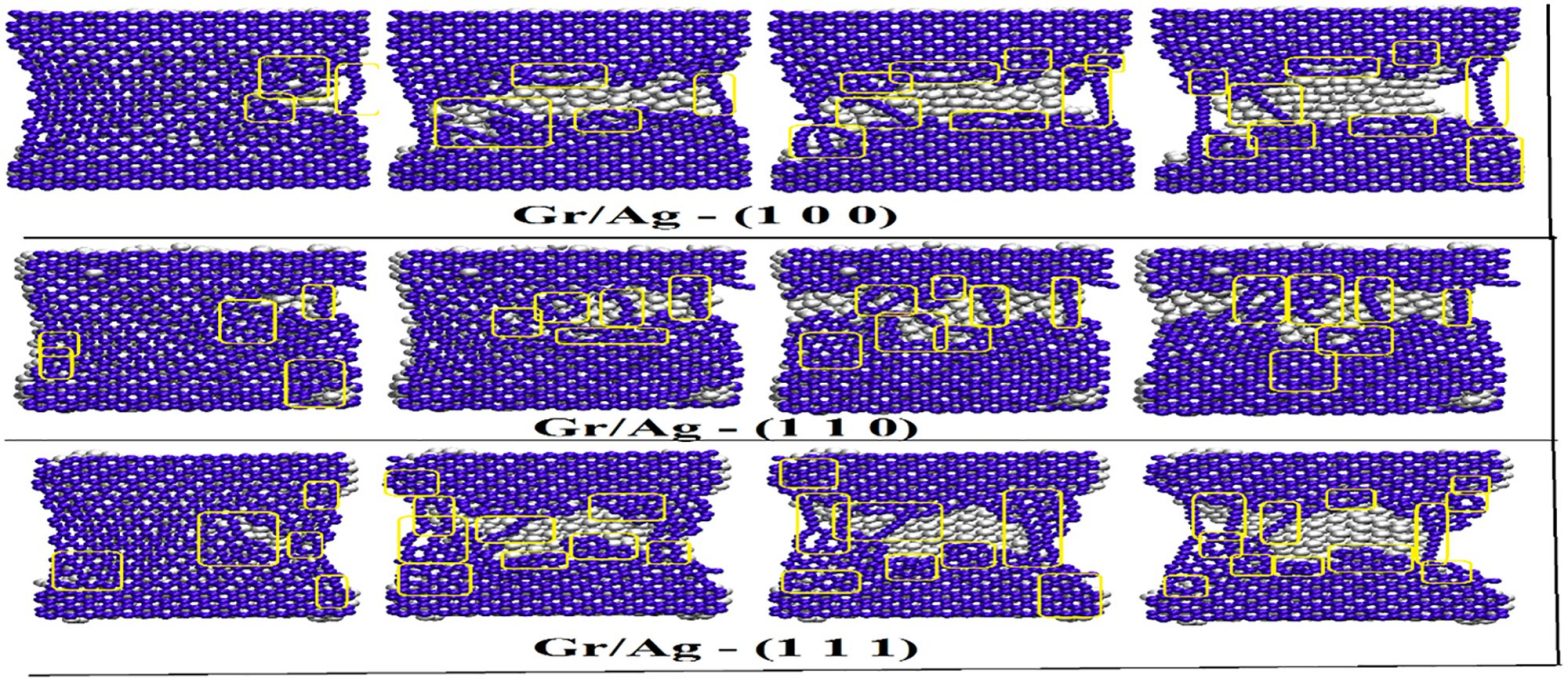
The mechanical characters of the Gr/Ag nano-composites along zigzag directions with respect to all three orientations. The boxes highlighted various types of dislocations.

**Fig 11:**
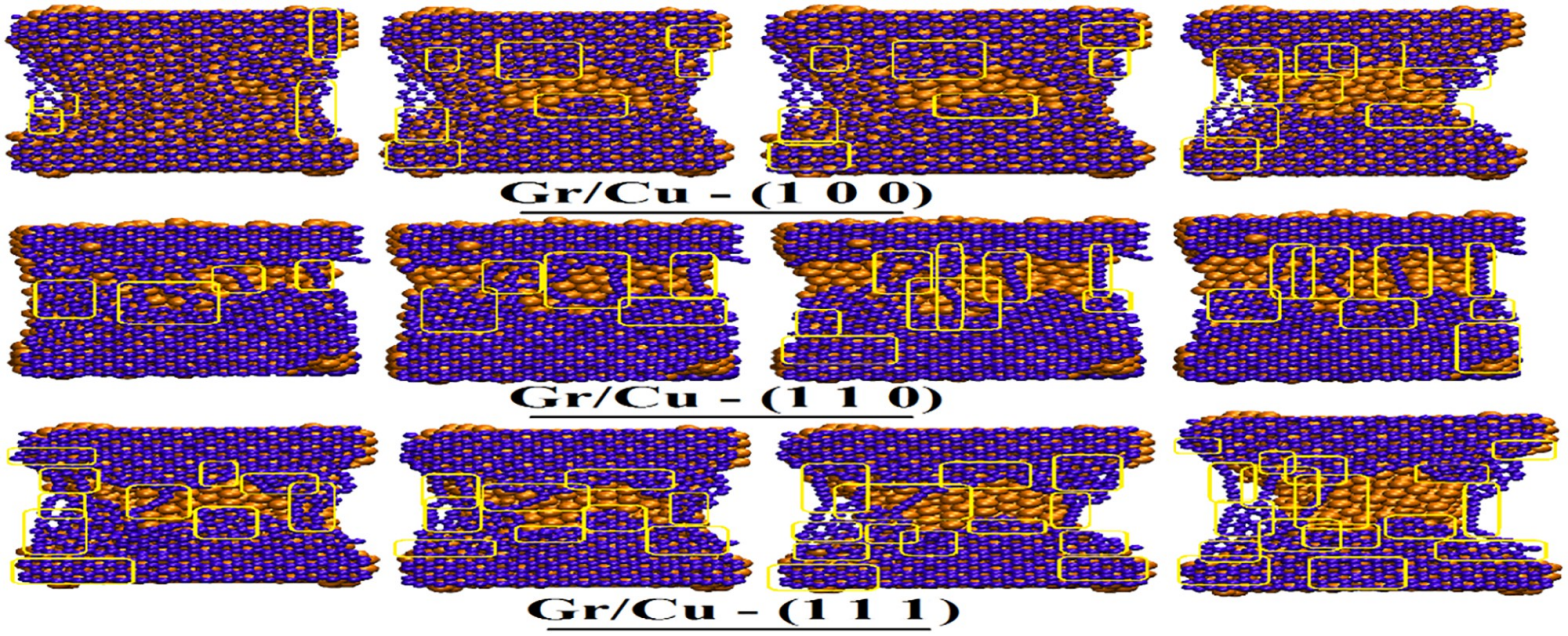
The mechanical characters of the Gr/Cu nano-composites along zigzag directions with respect to all three orientations. The boxes highlighted various types of dislocations.

The Figs 10 and 11 provide the fracture developments of Gr/Ag and Gr/Cu nano-composites, respectively, with respect to all considered orientations of metal substrate along zigzag directions. One may notice ductile fracture behavior along zigzag direction where breakage initiates at the sides of the nano-composites. Also, the mechanical characters are more evident along zigzag direction than arm-chair direction. A comprehensive comparative view of the Fig 10 clearly reveals the superior mechanical characters of Gr/Ag (1 1 1) dimension in comparison with the Gr/Ag (1 0 0) and Gr/Ag (1 1 0) orientations. Similarly, Fig 11 comprehends the mechanical attributes of the Gr/Cu nano-composites along zigzag directions. The strengthening behavior of Gr/Cu (1 1 1) outperforms the competitors. Consistently, once again Gr/Cu (1 1 1) projects most unique and attractive features when comparing both Gr/Metals nano-composites while considering all the taken orientations. The vividly more numbers of produced dislocations observable in the snapshots verify the advocated characteristics. Again, these findings are excellent match with the numerical findings reported in above sections.

## 4. Conclusion

The comparative evaluation of the thermo-mechanical characters of Gr/Ag and Gr/Cu layered structures is conducted. A rigorous application of the molecular dynamic simulation of the soft of LAMMPS is adopted to mimic the broad range of experimental conditions. Also, the thermo-mechanical attributes are enumerated with respect to varying dimensions such as (1 0 0), (1 1 0) and (1 1 1), of the metal substrates. Throughout the study, it is seen that the various orientations play driving role in the determination of the thermo-mechanical characteristics of both Gr/Metals structures. The (1 1 1) orientation signifies the most attractive features such as highest melting ladder and extravagant mechanical strength along both arm-chair and zigzag directions with respect to both Gr/Ag and Gr/Cu nano-composites. It is estimated that Gr/Ag (1 1 1) planes shows an increase of 5% in melting temperature as compared to its closest contemporary. Also, Gr/Cu (1 1 1) reveals an increased melting temperature of 11% in comparison to the close rival. Furthermore, while comparing leading (1 1 1) orientations across both Gr/Ag and Gr/Cu materials, the increased melting point is associated with Gr/Cu (1 1 1) dimension. In case of mechanical strength, both across arm-chair and zigzag directions, Gr/Metals (1 1 1) planes project elite attributes among the competing nano-composites. The maximum value of stress along arm-chair direction is estimated with respect to Gr/Cu (1 1 1) orientation showing numeric of 26.8 G Pa. Similarly, highest value of strain along arm-chair direction is estimated to be associated with Gr/Cu (1 1 1) indicating the value of 0.37. Further, zig-zag direction also witnessed enhanced mechanical strength associated with Gr/Cu (1 1 1) dimension. The findings reveal a value of 31 G Pa and numeric of 0.39 related to Gr/Cu (1 1 1) plane. The stress-strain curves are produced to assists the prediction of the isotopy of various orientations. The unique patterns of glide dislocation and shuffled dislocations. In general, it is well recognized in the literature that the generation of dislocations leads composites’ hardness. These findings are excellent advocacy of the inherent characters of these hybrid metallic Graphene structures.

The extravagant features of the novel Graphene have already earned a prime candidature in composite-focused research circles. However, production of the substrate at commercial level is still a watershed. It will be interesting to explore the characters of defective Graphene/Metals nano-composites to assist the notion of optimality. This can be considered an interesting future research venue. Also, the inclusion of other planes or direction remains an attractive research pursuit as the concentration of nuclei is planes sensitive. The growth of Gr crystal is determined by the flow of carbon fragments which then depends on the surface energy of the planes. Moreover, building on the findings of the simulation study, the replica of explored Gr/Metals nano-composites in experimental conditions will be a plausible future research direction. It is anticipated that composite materials having Gr particulates can be fabricated with respect to varying weight percent of Gr using stir casting method.
